# Perceived support and perceived ability promote walking behavior in Chinese adults with chronic diseases: the mediating role of health literacy based on the COM-B model

**DOI:** 10.3389/fpubh.2025.1676192

**Published:** 2025-12-03

**Authors:** Xue Tang, Xiaohua Zhong, Tian Liu, Guiqian Shi, Kangjie Li, Xinjing Liu, Cong Zhang, Xiaoni Zhong

**Affiliations:** 1 Chongqing Medical University College of Public Health, Chongqing, China; 2 Sichuan International Studies University, Chongqing, China

**Keywords:** health literacy, walk behavior, chronic diseases, perceived social support, general self-efficacy

## Abstract

**Background:**

Chronic diseases significantly contribute to global physical disability and mortality. Moderate-intensity aerobic activities, such as walking, are vital for managing chronic conditions. Research has shown that enhancing health literacy (HL) is crucial for promoting physical activity. Perceived social support (PSS) and general self-efficacy (GSE) affect HL. However, the effects of PSS and GSE on walking in patients with chronic diseases remain unclear. This study, grounded in the Capability, Opportunity, and Motivation-Behavior (COM-B) model, aimed to investigate the effects of PSS, GSE, and HL on walking in patients with chronic diseases.

**Methods:**

A total of 1,550 Chinese patients with chronic diseases were included in the analysis from the 2021 Chinese Residents’ Psychological and Behavioral Survey (PBICR), a cross-sectional survey. A multistage sampling approach was implemented at both provincial and municipal levels, complemented by quota sampling based on gender, age, and urban–rural distribution. Data were collected using a structured questionnaire, the Health Literacy Scale Short Form, the New General Self-Efficacy Scale, and the Perceived Social Support Scale. Descriptive and correlation analyses were conducted with SPSS 26.0. The mediating effect of HL on the relationship between PSS, GSE, and walking was analyzed with AMOS 21.0.

**Results:**

HL (*β* = 0.198, *p* < 0.001) significantly influenced walking. HL entirely mediated the effect of GSE on walking [*β* = 0.060, 95% CI = (0.040–0.082)]. HL partially mediated the effect of PSS on walking [*β* = 0.043, 95% CI = (0.026–0.061), effect size ratio = 21.50%].

**Conclusion:**

HL is crucial for promoting walking in patients with chronic diseases. Enhancing HL through PSS and GSE effectively promotes walking in individuals with chronic diseases.

## 1 Background

Chronic diseases contribute to physical disability and mortality, burdening global health and the economy (1). Moderate-intensity aerobic exercises, such as walking, are crucial for managing chronic disease symptoms (2). Walking improves cardiovascular health (3), cardiopulmonary and metabolic function (4), alleviates lower back pain (5), and reduces mortality risk (6). A study published in The Lancet Public Health found that, compared with walking 3,553 steps per day, walking 10,901 steps per day lowers the risk of death by 40–53% (7). Walking is a patient-friendly activity for individuals with chronic diseases. Walking does not require special equipment or facilities; it can be integrated into daily routines, such as during commutes or after meals. Strategies to promote walking are essential for improving the physical function of patients with chronic diseases.

Enhancing health literacy (HL) is critical for promoting physical activity (8). The Chinese version of the Health Literacy Scale (9) evaluates both the ability to seek (e.g., “identifying activities beneficial to mental health”), understand (e.g., “understanding media information about how to become healthier”), and evaluate health-related information (e.g., “assessing the impact of daily behaviors such as exercise and diet on health”), as well as the willingness to adopt health-related behavior (e.g., “if willing, joining a sports organization or fitness class”). This indicates that HL is a multidimensional structure that includes the ability to acquire, understand, and evaluate health-related information, and maps the motivation for adopting health-related behaviors.

**Figure 1:**
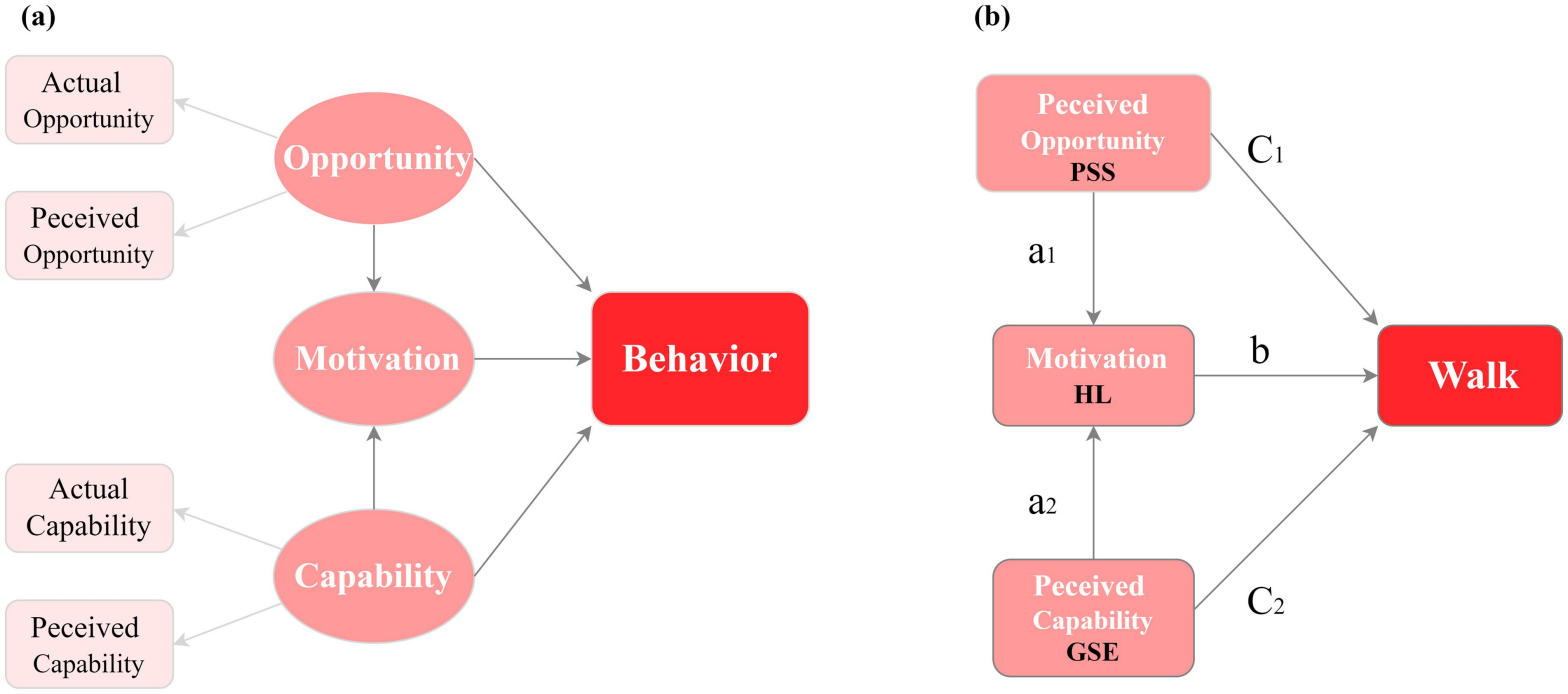
The COM-B model **(A)** and the hypothetical model **(B)**.

The Capability, Opportunity, and Motivation-Behavior (COM-B) model provides a framework for understanding behavioral changes (Figure 1A). In this model, “capability” encompasses the prerequisites for finishing a specific activity, while “opportunity” involves external factors that influence the individual. Capability and opportunity are crucial for motivation. When individuals have the capability, opportunity, and motivation, they are more inclined to engage in goal-directed behavior (10). Previous studies have investigated physical activity promotion strategies across various populations using the COM-B model (11, 12). Yang et al. identified the barriers and facilitators of exercise adherence in older adults using the COM-B model (11).

Previous studies have confirmed that motor skills (13) and social support (14) significantly improve physical activity levels. Nonetheless, no research has explored how perceived capability and perceived opportunity affect HL and walking of patients with chronic diseases from a sociopsychological perspective grounded in the COM-B model. General self-efficacy (GSE) is a person’s belief in their own abilities (15). It is about how people see their skills from a psychological view and is linked to Capability in the COM-B model. Perceived social support (PSS) refers to an individual’s perceived level of accessible social capital (16). PSS reflects perceived opportunities to practice health-related behaviors. Previous research has confirmed that GSE and PSS enhance HL and physical activity (15, 17–21). We speculate that PSS may encompass perceived opportunities for walking; GSE may encompass perceptions of one’s walking-related abilities; and HL may encompass knowledge of the benefits of walking, intention, and motivation to walk. This study uses the COM-B framework to hypothesize that PSS (seen as opportunity), GSE (seen as capability), and HL (linked to motivation for walking) all help patients with chronic diseases walk more. PSS and GSE affect the walking behavior of patients with chronic diseases through HL (Figure 1B).

China provides an advantageous context for studying walking behavior in patients with chronic diseases. As the country with the largest number of patients with chronic diseases in the world, in the past decade, China has initiated walking campaigns in 1,106 chronic disease prevention zones across 31 provinces, encouraging residents to walk 10,000 steps every day^1^ (22). Therefore, we conducted a study among the Chinese population to provide evidence for promoting walking behavior in patients with chronic diseases globally. Our hypotheses include:

*H1*: HL directly enhances walking in individuals with chronic diseases.

*H2*: PSS promotes walking in individuals with chronic diseases by HL.

*H3*: GSE promotes walking in individuals with chronic diseases by HL.

## 2 Methods

### 2.1 Study design

This study was reported in accordance with the STROBE guidelines for cross-sectional studies. The data utilized were acquired upon application from the 2021 China Psychological and Behavioral Survey (PBICR). It covered 23 provinces, four municipalities, and five autonomous regions in mainland China. A multistage sampling approach was implemented at both provincial and municipal levels, complemented by quota sampling based on gender, age, and urban–rural distribution. The PBICR received approval from the Ethical Review Committee (approval numbers: JKWH-2021-01 and JNUKY-2021-018), and informed consent was obtained from all participants, for details on the PBICR’s design, methodology, survey, and data access.^2^

### 2.2 Participants

**Figure 2:**
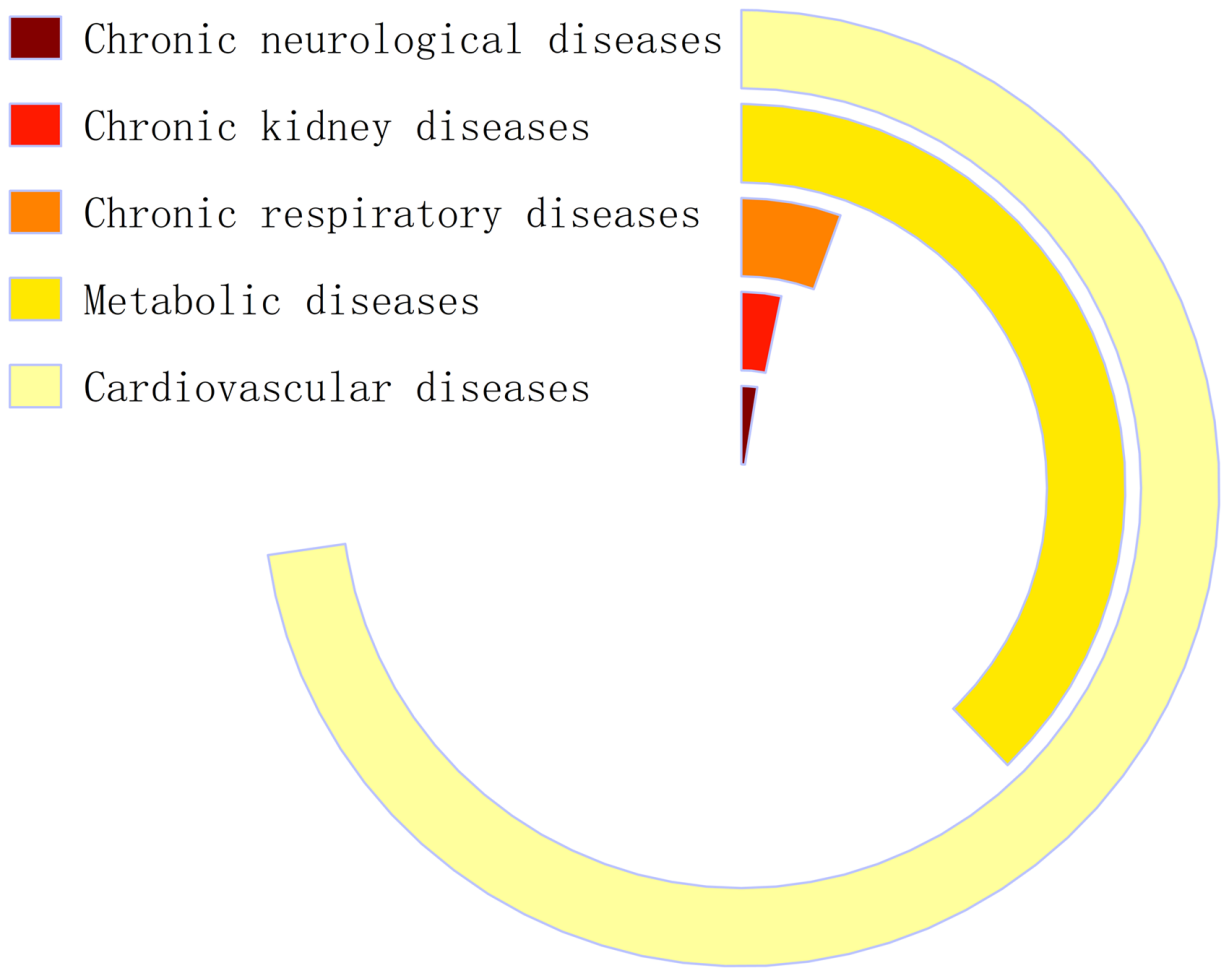
Composition of chronic disease types among participants.

The study population comprised adult patients diagnosed with chronic diseases in the PBICR. Inclusion criteria: Patients with chronic diseases for which moderate-intensity physical activity is recommended as a symptom management strategy by guidelines or expert consensus. Patients with cardiovascular and cerebrovascular diseases (e.g., hypertension and coronary heart disease), metabolic disorders (e.g., diabetes, fatty liver disease, and dyslipidemia), chronic respiratory diseases (e.g., chronic obstructive pulmonary disease and asthma), chronic neurological disorders (e.g., Alzheimer’s and Parkinson’s diseases), and chronic kidney disease were included. The exclusion criteria were chronic diseases (chronic gastritis and chronic enteritis), which did not include walking as a symptom control and improvement intervention in the guidelines. Some tumors have also been included in the category of chronic diseases for management. There are differences in symptom management methods for patients with tumors at different stages. Patients with early- or mid-stage cancer might benefit from exercise to help their immune system. However, this may not be suitable for those with late-stage cancer. Patients with tumors were excluded because this study did not investigate the treatment period for those patients. Some participants simultaneously suffered from multiple chronic diseases. The study could not analyze the samples by weight because there was no information on the gender, age, or location of people with multiple chronic diseases in 2021. A total of 1,550 patients with one or more chronic diseases were enrolled. Among them, 1,128 had cardiovascular disease, 586 had metabolic disease, 39 had chronic neurological disease, 51 had chronic kidney disease, and 86 had chronic respiratory disease (Figure 2). The findings are most generalizable to patients with cardiovascular and metabolic conditions.

### 2.3 Measurement

This study used a standardized questionnaire to assess participants’ demographic characteristics, including gender, age, education level, and average monthly per capita household income.

#### 2.3.1 Walking behavior

The measurement results of residents’ walking behavior levels were obtained from the PBICR 2021 survey questionnaire. In the questionnaire, participants were asked, “Over the past week, how much time did you spend on walking?” Response was assigned a score on a scale from 0 to 4, corresponding to the categories “none,” “less than 30 min,” “30 ~ 59 min,” “1 ~ 3 h,” and “more than 3 h.” The total score ranged from 0 to 4, with higher scores indicating a longer walking duration over the past week.

#### 2.3.2 Health literacy

The Chinese version of the Health Literacy Scale Short-Form (HLS-SF12) (9) includes 12 items across three dimensions: health care, disease prevention, and health promotion. This study investigated the dimensions of health promotion. This dimension contains items like “Identifying activities that are beneficial to your mental health (e.g., meditation, exercise, walking, Pilates).” “Understanding health-related information presented in media sources (e.g., the internet, newspapers, magazines) regarding how to improve personal health.” “Evaluating which daily behaviors (e.g., alcohol consumption, dietary habits, physical activity) may influence your health.” And “Willingness to participate in a sports organization or fitness class, if interested.” A 4-point Likert scale was used to produce scores ranging from 4 to 16. Higher scores indicate better HL. The scale showed high internal consistency (Cronbach’s *α* = 0.94).

#### 2.3.3 General self-efficacy

General self-efficacy is defined as the perception of an individual’s overall ability to perform across various situations. In this study, the New General Self-Efficacy Scale (NGSES) by Chen et al. (23) was used. This scale includes eight items and uses a 5-point Likert scale, with responses ranging from 1 (strongly disagree) to 5 (strongly agree). The cumulative score ranges from 8 to 40, with higher scores indicating greater GSE. The scale showed high reliability (Cronbach’s *α* = 0.94).

#### 2.3.4 Perceived social support

Perceived social support, defined as the perception of available support from a social network, was assessed using the widely used Chinese version of the Perceived Social Support Scale (16). The scale comprises 12 items that measure perceived social support from families, friends, and other sources. Responses were scored on a 7-point Likert scale, with total scores ranging from 12 to 84. Higher scores indicate a greater level of PSS, with high internal consistency (Cronbach’s *α* = 0.92).

### 2.4 Data analysis

Data analysis was performed using SPSS version 26.0 and AMOS version 21.0. All study variables exhibited normal distributions. The main objective of this study was to examine the path relationships in the structural model. The survey in this study used a 5-point scale to rate walking. Its distribution was approximately normal with a sufficiently large sample size; therefore, we treated it as a continuous variable. Descriptive statistics included the means (standard deviations) and frequencies (percentages) of the variables. Pearson correlation coefficients were used to clarify the relationships between the variables. The variance inflation factor tests showed that all values were below 2, confirming no multicollinearity among the variables. The mediation effects were tested using the bootstrap method with 5,000 resamples. The models were adjusted for covariates, including gender, age, education, and household monthly income per capita.

## 3 Results

### 3.1 Characteristics of the participants

Table 1 presents the fundamental demographic characteristics of the participants. The sample comprised 1,550 individuals with chronic diseases, of whom 77.29% (*n* = 1,198) were over 46 years old. Additionally, 72.00% (*n* = 1,116) reported a monthly per capita household income below 6,000 yuan, and 45.61% (*n* = 707) attained an education level of junior high school or lower. Table 2 summarizes the walking and related variables. The mean walking score was 2.44 ± 1.35 (range: 0–4), the mean HL score was 11.56 ± 2.16 (range: 4–16), the average GSE score was 28.35 ± 5.26 (range: 8–40), and the mean PSS score was 59.76 ± 12.02 (range: 12–84).

**Table 1:** Fundamental characteristics of participants (*N* = 1,550).

Variable	n	%
Gender		
Male	871	56.20
Female	679	43.80
Age		
~45	352	22.71
46 ~ 59	559	36.06
60~	639	41.23
Monthly household income per capita (yuan)		
≤3,000	492	31.74
3,001 ~ 6,000	624	40.26
6,001~	434	28.00
Education		
9 years—Junior high school or lower	707	45.61
12 years—high school	309	19.94
15 years—Junior college or higher	534	34.45

**Table 2:** Descriptive analysis of variables (*N* = 1,550).

Variable	Walking	GSE	PSS	HL
Mean	2.44	28.35	59.76	11.56
SD	1.35	5.26	12.02	2.16
Min	0.00	8.00	12.00	4.00
Max	4.00	40.00	84.00	16.00

### 3.2 Correlation analysis

**Figure 3:**
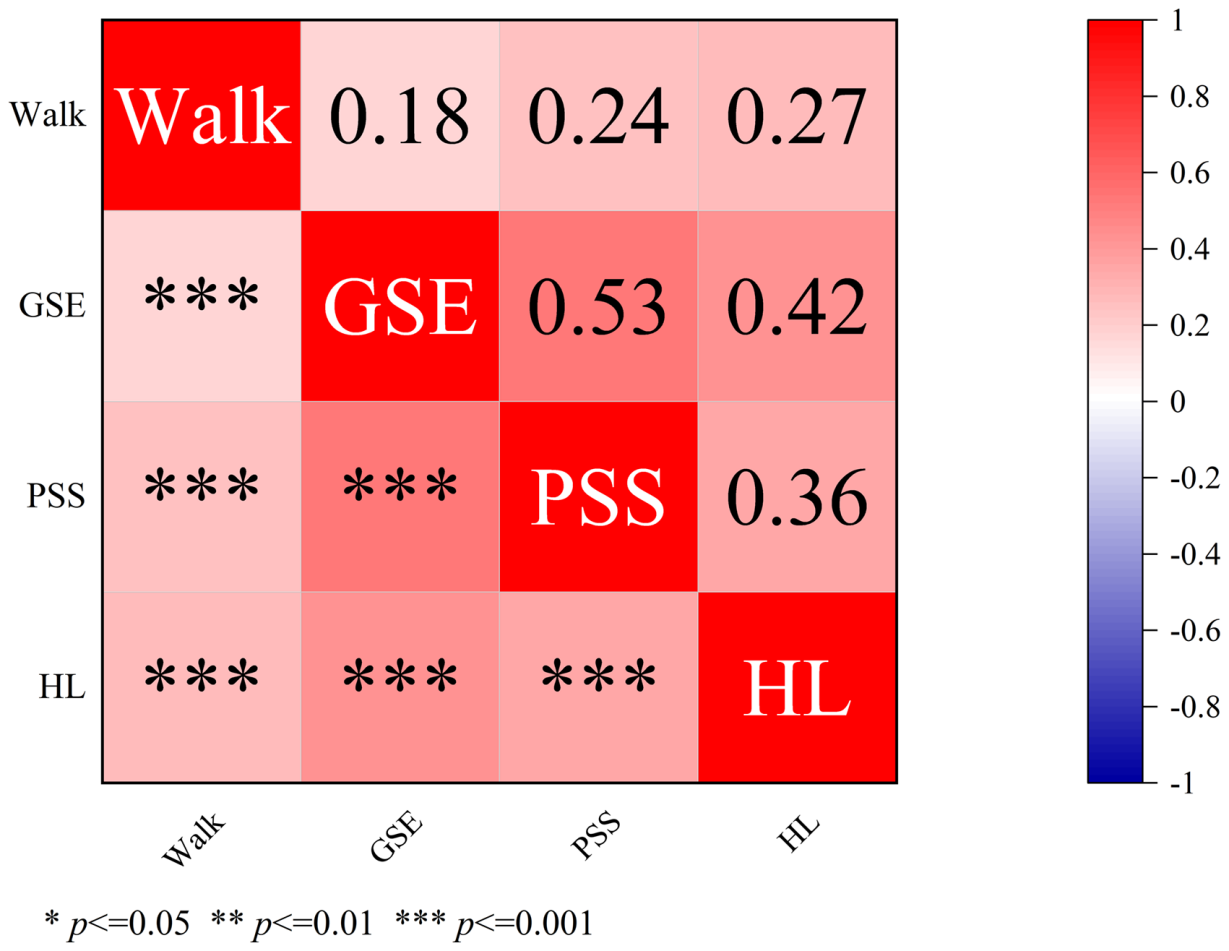
Correlations among the main study variables (*N* = 1,550).

Figure 3 shows the relationships between the PSS, GSE, HL, and walking. The results showed that GSE, PSS, and HL were all associated with walking behavior. HL had the strongest link (*r* = 0.27, *p* < 0.001), followed by PSS (*r* = 0.24, *p* < 0.001) and GSE (*r* = 0.18, *p* < 0.001). GSE and PSS showed a moderately significant positive correlation with HL, with GSE showing a higher correlation with HL (*r* = 0.42, *p* < 0.001) than PSS (*r* = 0.36, *p* < 0.001).

### 3.3 Mediating effect

**Figure 4:**
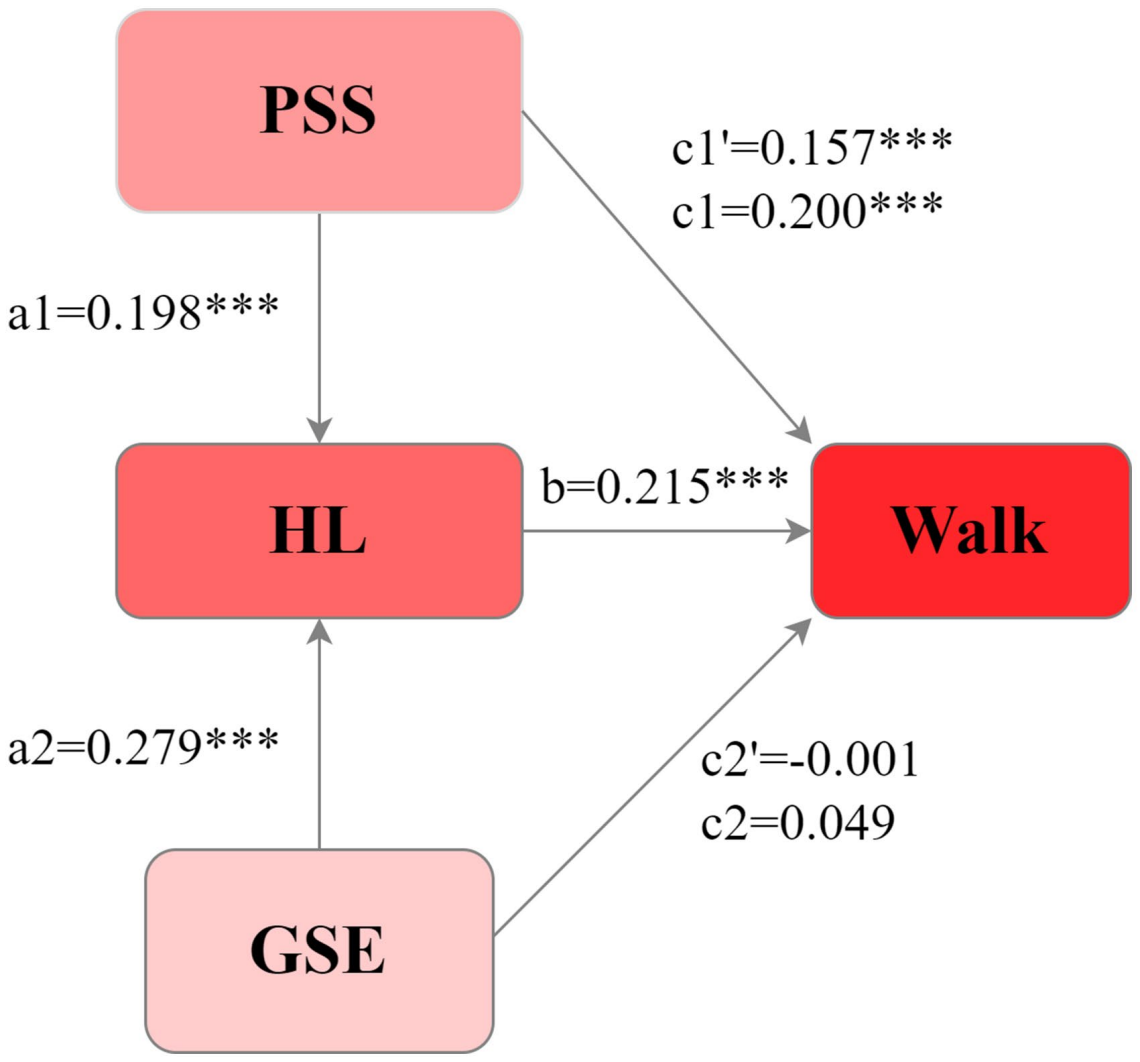
Mediation model of HL on the relationship between PSS, GSE, and walking in patients with chronic diseases (data are standardized coefficients).

Table 3 shows that HL mediated the relationship between PSS, GSE, and walking behavior. Figure 4 shows the results of the mediation analysis. The data and the proposed conceptual model matched well (CFI = 0.995, TLI = 0.951, SRMR = 0.018, and RMSEA = 0.048, χ^2^/df = 4.63).

**Table 3:** Standardized effects of the mediating model of walking (*N* = 1,550).

Path	*Sβ*	Boot SE	95%CI		*P*
			Lower	Upper	
PSS → HL	0.198	0.030	0.137	0.254	<0.001
GSE → HL	0.279	0.035	0.210	0.346	<0.001
HL → Walk	0.215	0.029	0.158	0.270	<0.001
PSS → HL → Walk	0.043	0.009	0.026	0.061	<0.001
GSE → HL → Walk	0.060	0.011	0.040	0.082	<0.001
PSS → Walk (direct effect)	0.157	0.029	0.103	0.214	< 0.001
PSS → Walk (total effect)	0.200	0.029	0.144	0.256	<0.001
GSE → Walk (direct effect)	−0.011	0.030	−0.070	0.049	0.725
GSE → Walk (total effect)	0.049	0.030	−0.011	0.110	0.105

HL entirely mediated the association between GSE and walking behavior. GSE significantly influenced HL [*β* = 0.279, 95% CI = (0.210–0.346), *p* < 0.001], and HL positively influenced walking [*β* = 0.215, 95% CI = (0.158–0.270), *p* < 0.001]. The total effect of GSE [*β* = 0.049, 95% CI = (−0.011–0.110), *p* = 0.105] and the direct effect [*β* = −0.011, 95% CI = (−0.070–0.049), *p* = 0.725] on walking were not significant. HL exhibited a significant indirect effect of GSE on walking [*β* = 0.060, 95% CI = (0.040–0.082), *p* < 0.001], suggesting that GSE indirectly influences walking in patients through HL mediation.

HL partially mediated the relationship between PSS and walking. The PSS positively affected HL [*β* = 0.198, 95% CI = (0.137–0.254), *p* < 0.001], and HL influenced walking [*β* = 0.215, 95% CI = (0.158–0.270), *p* < 0.001]. The total [*β* = 0.200, 95% CI = (0.144–0.256), *p* < 0.001] and direct [*β* = 0.157, 95% CI = (0.103–0.214), *p* < 0.001] effects of PSS on walking were significant. The indirect effect of HL on the relationship between PSS and walking was significant [*β* = 0.043, 95% CI = (0.026–0.061), *p* < 0.001], accounting for 21.50% of the total effects. This suggests that PSS can directly promote walking and, indirectly, promote walking in patients with chronic diseases through HL.

## 4 Discussion

This study, grounded in the COM-B framework, explored the effects of GSE and PSS on HL and walking behavior in patients with chronic diseases. Although cross-sectional studies limit causal inference, this study has implications for guiding walking behavior interventions in patients with chronic diseases. Mediation analysis revealed that HL directly promoted walking in patients. PSS and GSE enhanced walking behavior in patients with chronic diseases via HL.

### 4.1 HL enhanced the walking behavior

Our study found that HL enhanced walking behavior in patients with chronic diseases, supporting the research hypothesis (H1). Nutbeam et al. (24) defined HL as the ability to read, understand, and evaluate health information to make behavioral decisions (25). Hence, HL includes knowledge, skills, intentions, and motivations related to health. Previous studies have shown that HL-based interventions significantly enhance walking behavior among healthy volunteers (26). Older adults with insufficient HL are 38% less likely to exercise five or more days/week than those with adequate HL (27). Individuals with adequate HL better understand the benefits of walking for managing chronic disease symptoms, leading to greater physical activity than those with insufficient HL. Whereas Al Sayah et al. found no significant link between daily steps and HL in older adults (28). The authors did not explain this issue. We hypothesized that this discrepancy may stem from the criteria used to classify HL as adequate. Al Sayah’s study found that 91.17% of individuals had adequate HL, while 23.15% nationwide in China in 2020 (29). Our findings, along with numerous prior studies (27, 29), indicate that HL is crucial for promoting physical activity.

### 4.2 PSS directly promotes walking and indirectly promotes walking through HL

Consistent with the research hypothesis (H2), we found that PSS enhanced the positive impact on walking behavior through HL and directly promoted walking among patients with chronic diseases. Social Capital refers to a collective resource comprising shared norms, beliefs, networks, and social connections (30). PSS refers to the perceived level of accessible social capital (16). Studies have revealed that PSS has a significant positive effect on mental HL (21) and overall e-HL (20). In the digital age, the Internet has become the primary channel for disseminating HL information. Governments and health agencies share health information and courses through the media and provide online health social capital to patients with chronic diseases (31–33). For example, walking campaigns in China that encourage staff and workers to walk 10,000 steps every day show that online social support (likes, comments, shares) can increase walking steps among Chinese residents the next day (22). Walking campaigns in China are public institution-based health promotion activities. We speculate that China’s collectivism shapes the PSS and HL of patients with chronic diseases in China. Walking campaigns turn healthy behavior into a shared task, fostering a sense of group pride that compensates for the limits of personal willpower. On the other hand, more than half of the patients with chronic diseases in the sample of this study were middle-aged and old adults. Out of filial piety, children and grandchildren in China often help older patients access online health resources and teach older adults how to use smart devices to track steps. Intergenerational digital support enhances older patients’ perceptions of the availability of online health information. Moreover, PSS can directly promote walking behavior in patients with chronic diseases. Previous studies have found that during the COVID-19 pandemic, individuals with higher PSS levels were 64% more likely to exercise than those with lower PSS levels (19). Social support from the closest person helped British men maintain the recommended level of physical activity (34).

### 4.3 GSE promotes walking by HL

Consistent with the research hypothesis (H3), GSE positively influenced walking behavior in patients with chronic diseases through HL. Bandura posited that self-efficacy is the primary cognitive mechanism for behavioral change. Self-efficacy is the belief in one’s ability to sustain behaviors to achieve desired outcomes (35). The study revealed a medium effect size (*β* = 0.279, *p* < 0.001) for GSE on HL, indicating that boosting GSE in patients with chronic diseases effectively promoted HL. Individuals with higher GSE are more confident in learning and practicing health-related behaviors than those with low GSE (15). Research has shown a positive correlation between GSE and HL in patients with diabetes and hypertension (36, 37). In Turkish women, Pap smear screening-related HL increased as their self-efficacy levels increased (38). Populations with high HL levels are more proactive in acquiring and applying health-related information (18), thereby promoting health-related behaviors (27, 39), including walking.

However, this study did not find a direct correlation between GSE and walking behavior in individuals with chronic conditions. This may be because HL was included as a mediator in this model. Our findings support Hubner et al. (40) study, which showed that GSE affects behavioral outcomes through cognitive variables. Hubner et al. found that the effect of the GSE on physical activity in patients after bariatric surgery was fully mediated by an internalized weight bias (40). In our study, HL with cognitive variables was used to promote walking exercise in patients with chronic diseases. This discovery emphasizes that GSE only affects the walking behavior of patients with chronic diseases by influencing HL.

### 4.4 Implications

In this model, HL had the strongest association with walking behavior (*β* = 0.215, *p* < 0.001). It was more influential than PSS (*β* = 0.200, *p* < 0.001) and GSE (*β* = 0.049, *p* > 0.05). Therefore, HL is crucial for promoting walking behavior among patients with chronic diseases. Specifically, this can be achieved by enhancing perceptions of opportunities to access and use online health resources. For example, community doctors could, based on the scenario, push regular notifications about the benefits of walking and related activities. When the weather forecast predicts rain, push indoor walking exercise video links and recommend nearby parks suitable for walking on weekends. This makes it easier for people to access health resources and walking opportunities. We can also boost their confidence, making them more likely to seek health information and to promote walking. For example, setting small walking goals, such as a 5-min daily walk, helps patients feel more in control of achieving their goals, boosting their confidence in their abilities. Subsequently, gradually increase the walking time. Encourage each other through peer encouragement, enhance self-efficacy, and ultimately promote walking behavior in patients with chronic diseases.

#### 4.4.1 Strengths and limitations

This study is the first to explore the effects of PSS, GSE, and HL on walking behavior in patients with chronic diseases using the COM-B model. The results suggest that perceived opportunities and capabilities shape walking behavior by influencing HL. This study had some limitations. The cross-sectional study design could not determine causality. Specific public health initiatives (such as walking campaigns in public institutions) and social support systems influenced by collectivist norms in China might shape the observed relationships, warranting caution when generalizing to other cultural settings. Given the substantial proportion of patients with cardiovascular and cerebrovascular diseases, as well as metabolic disorders, in the sample population of this study. The findings are representative of individuals with these conditions. Consequently, the results may not apply to patients with other chronic diseases. Self-reported walking behavior may be influenced by recall and social-expectation biases. Future longitudinal studies using pedometer tools are essential to understand walking behavior in individuals with chronic diseases.

## 5 Conclusion

HL mediates the effects of PSS and GSE on walking. Enhancing HL may be a key strategy for increasing walking among individuals with chronic diseases. Future longitudinal studies using pedometer tools and intervention-based studies are warranted.

## Data Availability

The datasets presented in this study can be found in online repositories. The PBICR’s data access, visit https://www.x-mol.com/groups/pbicr/.

## Footnotes

1
www.wanbu.com.cn

2
https://www.x-mol.com/groups/pbicr/
